# Use of Rotterdam Risk Index in the Assessment of Risk Factors in Abdominal Wound Dehiscence

**DOI:** 10.7759/cureus.107456

**Published:** 2026-04-21

**Authors:** Likith Y A, Girish Kullolli, Tejaswini Vallabha, Shruti Sheelin, Manjunath Savant, Snehal Patil

**Affiliations:** 1 General Surgery, Shri B M Patil Medical College, Hospital and Research Centre, Bijapur Lingayat District Educational Association (BLDE), Vijayapura, IND

**Keywords:** abdominal wound dehiscence, laparotomy, risk factors, rotterdam risk index, surgical site infection

## Abstract

Introduction: Abdominal wound dehiscence (AWD) is a serious postoperative complication characterized by partial or complete disruption of a surgical incision, often leading to increased morbidity, prolonged hospitalization, and higher healthcare burden. Its occurrence is multifactorial, involving patient-related, surgical, and postoperative factors. Risk assessment tools such as the Rotterdam Risk Index (RRI) have been used to evaluate the likelihood of AWD; however, as the index includes postoperative variables, it reflects post-hoc risk stratification rather than purely preoperative prediction.

Materials and methods: A prospective observational study was conducted over 22 months in a tertiary care center, including 70 patients undergoing abdominal midline laparotomies. Patients undergoing relaparotomy were excluded. Clinical, surgical, and postoperative variables were recorded. The primary outcome was the occurrence of AWD, while secondary outcomes included wound infection and its association with RRI. The RRI, incorporating preoperative, intraoperative, and postoperative variables, was calculated during the postoperative period. Analysis was performed using bivariate methods. Statistical analysis was performed using SPSS software, version 26 (IBM Corp., Armonk, NY), and Receiver Operating Characteristic (ROC) curve analysis was used to assess predictive accuracy.

Results: The mean age was 46.66 ± 18.46 years, with male predominance (39, 55.7%). AWD occurred in 8 (11.4%) patients, while wound infection was observed in 29 (41.4%). Significant risk factors for AWD included COPD (4, 33.3%, p = 0.003), postoperative cough (2 (100.0%) p < 0.001), type of surgery (p = 0.049), and wound infection (6 (20.7) vs 1 (2.4%), p = 0.012). The mean RRI score was significantly higher in patients with AWD (6.58 ± 0.84) compared to those without (3.95 ± 1.36) (p < 0.001). ROC analysis demonstrated excellent predictive accuracy (Area Under the Curve (AUC) = 0.956), with an optimal cut-off ≥5.8 yielding 90% sensitivity and 92% specificity. The positive predictive value (PPV) and negative predictive value (NPV) were 59% and 98%, respectively.

Conclusion: AWD is influenced by multiple clinical and surgical factors. Postoperative and surgical factors showed stronger associations in this study. The Rotterdam Risk Index demonstrated good discriminative ability; however, as it incorporates postoperative variables, it reflects association rather than early prediction. Further validation using preoperative models is required.

## Introduction

Surgical wound dehiscence (SWD) is defined as the partial or complete disruption of a previously closed surgical incision and may involve superficial layers or extend to deeper fascial planes [1]. It represents a serious postoperative complication with a variable clinical presentation depending on the depth and underlying cause [2]. While some cases are associated with overt infection, others may occur in the absence of clear clinical signs, making early detection challenging [1]. Among its various forms, abdominal wound dehiscence (AWD) is particularly significant due to its potential to compromise fascial integrity and lead to severe morbidity [3].

AWD continues to be a major concern in surgical practice, with reported incidence ranging from 0.4% to 3.5% and mortality rates reaching up to 45% [4,5]. It is associated with a prolonged hospital stay, increased need for reoperation, and substantial healthcare burden [6]. The etiology of AWD is multifactorial, encompassing patient-related factors such as anemia, nutritional deficiencies, and comorbidities; surgery-related factors such as emergency procedures, wound contamination, and technical aspects of closure; as well as postoperative factors like surgical site infection and increased intra-abdominal pressure [7,8]. The complex interplay of these variables highlights the need for comprehensive risk assessment in surgical patients [8].

In response to this, several risk prediction models have been developed to aid in the risk assessment of patients undergoing abdominal surgery. The Rotterdam Risk Index (RRI), introduced in 2010, integrates multiple clinical variables into a composite score to estimate the probability of abdominal wound dehiscence [9]. The RRI includes variables such as age, sex, anemia, chronic obstructive pulmonary disease (COPD), ascites, jaundice, emergency surgery, type of surgery, postoperative cough, and wound infection [9]. Previous studies have demonstrated that higher RRI scores are associated with a significantly increased risk of dehiscence, supporting its association with increased risk of dehiscence [10]. Its simplicity and applicability make it particularly attractive for routine surgical practice.

Despite its demonstrated utility, the Rotterdam Risk Index remains underutilized in many clinical settings, particularly in resource-limited environments. Moreover, there is a need to validate its predictive performance across diverse patient populations and to better understand its association with commonly encountered clinical risk factors. In view of these considerations, the present study aimed to evaluate the risk factors associated with abdominal wound dehiscence and to assess the association and discriminative ability of the Rotterdam Risk Index in relation to AWD, acknowledging that the index includes postoperative variables and therefore reflects post-hoc risk stratification rather than purely preoperative prediction. No formal hypothesis was predefined, and the study was exploratory in nature. This research work was originally conducted as part of a postgraduate dissertation submitted to the Department of General Surgery, BLDE (Deemed to be University), Vijayapura, Karnataka, India.

## Materials and methods

A prospective observational study was conducted in the Department of General Surgery at Shri B M Patil Medical College Hospital and Research Centre, Bijapur Lingayat District Educational Association (BLDE), Vijayapur, over a period from March 2024 to December 2025. All consecutive patients admitted during this period who underwent abdominal midline laparotomies were included using a consecutive sampling technique. During the study period, a total of 92 laparotomies were performed, of which 70 patients met the inclusion criteria and were enrolled. Institutional ethical clearance was obtained prior to the study (IEC No: BLDE(DU)/IEC-SBMPMC/097/2023-24), and written informed consent was obtained from all participants.

The sample size was calculated based on an anticipated incidence of abdominal wound dehiscence ranging from 0.3% to 3.5%, with a 95% confidence level and 4% absolute precision using the formula n = Z²pq/d², where Z represents the standard normal deviate at the desired level of significance, p the expected proportion, q = 100−p, and d the allowable error [5]. Based on this calculation, a minimum sample size of 70 patients was obtained, and all eligible patients were included in the study.

Patients aged 18 years and above undergoing abdominal midline laparotomies, both elective and emergency, were included. Patients undergoing relaparotomy and those with known collagen vascular diseases or connective tissue disorders were excluded. Patients undergoing relaparotomy were excluded to avoid confounding due to altered wound healing dynamics and a higher baseline risk of dehiscence. A detailed clinical history was recorded, and a comprehensive physical examination was performed for each patient. Preoperative optimization was carried out as required, including correction of anaemia and management of comorbidities such as hypertension and diabetes mellitus.

All patients underwent standardized continuous fascial closure of the linea alba using non-absorbable monofilament synthetic suture (polypropylene No. 1). Data regarding suture-to-wound length ratio, bite size, and interval distance were not recorded. Closure was performed by resident surgeons under the supervision of attending consultants. Retention sutures were not routinely used. Antibiotic prophylaxis was administered in all elective cases, while patients presenting with an acute abdomen received extended antibiotic therapy based on intraoperative findings. Postoperative wound assessment was initiated from the third postoperative day and continued until suture removal and early postoperative recovery. Wounds were evaluated for clinical signs, including erythema, edema, discharge, and any evidence of disruption.

The primary outcome of the study was the occurrence of abdominal wound dehiscence, defined as partial or complete disruption of the abdominal wound involving fascial separation, with or without evisceration [11]. Secondary outcomes included the occurrence of wound infection and its association with the Rotterdam Risk Index. Wound infection was defined based on clinical evidence of purulent discharge, erythema, or microbiological confirmation [12].

Each patient was assessed using the Rotterdam Risk Index, which incorporates preoperative, intraoperative, and postoperative variables. The score was calculated during the early postoperative period (after postoperative day 3 and prior to discharge) using predefined criteria. The components of the RRI included age, sex, anemia, COPD, ascites, jaundice, emergency surgery, type of surgery, postoperative cough, and wound infection. The probability of abdominal wound dehiscence was estimated using the logistic regression equation P = ex / (1 + ex) × 100, where x = -8.37 + (1.085 × total risk score) [9]. The predicted probability was then compared with the actual clinical outcome. As the RRI includes postoperative variables, its use in this study reflects association rather than purely preoperative prediction. Outcome assessors were not blinded to RRI scores.

All data were entered into Microsoft Excel and analyzed using the Statistical Package for the Social Sciences (SPSS), version 26 (IBM Corp., Armonk, NY). Continuous variables were expressed as mean ± standard deviation and analyzed using an independent t-test. Categorical variables were presented as frequencies and percentages and analyzed using appropriate statistical tests (Chi-square test or Fisher’s exact test, as applicable based on expected cell counts). Receiver operating characteristic (ROC) curve analysis was performed to assess the predictive accuracy of the Rotterdam score and to determine the optimal cut-off value using the Youden index. A p-value of less than 0.05 was considered statistically significant. Only bivariate analysis was performed; multivariate analysis was not undertaken.

## Results

The study included 70 patients with a mean age of 46.66 ± 18.46 years, predominantly in the 40-49 years group (14, 20.0%). Males constituted 39 (55.7%) of the cohort. Among comorbidities, anemia was the most common (39, 55.7%), followed by COPD (12, 17.1%) and jaundice (8, 11.4%), while ascites (5, 7.1%) and cough (2, 2.9%) were less frequent (Table 1).

**Table 1:** Baseline characteristics (n = 70) This table presents the baseline demographic and clinical characteristics of the study population (n = 70). Age is categorized into groups and expressed along with sex distribution and clinical comorbidities. Data presentation: Categorical variables are expressed as frequency (n) and percentage (%). Statistical test used: Descriptive statistics only; no inferential test applied. n – Number of patients, % – Percentage, COPD – Chronic Obstructive Pulmonary Disease.

Variable	Category	N (%)
Age Group (years)	18–19	5 (7.1%)
	20–29	10 (14.3%)
	30–39	10 (14.3%)
	40–49	14 (20.0%)
	50–59	11 (15.7%)
	60–69	10 (14.3%)
	70–85	10 (14.3%)
Sex	Male	39 (55.7%)
	Female	31 (44.3%)
Clinical Profile	Anemia	39 (55.7%)
	Ascites	5 (7.1%)
	COPD	12 (17.1%)
	Cough	2 (2.9%)
	Jaundice	8 (11.4%)

Peptic ulcer perforation was the most common preoperative diagnosis (18, 25.7%), followed by small bowel obstruction and trauma abdomen (10, 14.3% each). Other notable causes included bowel perforation/gangrene (7, 10.0%) and hernia (6, 8.6%), while remaining conditions were relatively less frequent (Table 2).

**Table 2:** Preoperative diagnosis This table summarizes the distribution of preoperative diagnoses among the study participants. Data presentation: Data are presented as frequency (n) and percentage (%). Statistical test used: Descriptive statistics only. n – Number of patients, % – Percentage.

Diagnosis	N (%)
Peptic ulcer perforation	18 (25.7%)
Small bowel obstruction	10 (14.3%)
Trauma abdomen	10 (14.3%)
Bowel perforation/gangrene (non-ulcer)	7 (10.0%)
Hernia	6 (8.6%)
Pancreatic pathology	4 (5.7%)
Malignancy (colorectal/ovarian)	4 (5.7%)
Volvulus	3 (4.3%)
Gallbladder pathology	1 (1.4%)
Miscellaneous	7 (10.0%)

Small bowel surgeries were most common (29, 41.4%]), followed by gastroduodenal (25, 35.7%) and large bowel procedures (13, 18.6%). Peptic ulcer repair was the most frequently performed procedure (14, 20.0%), followed by resection and anastomosis (11, 15.7%) and adhesiolysis (9, 12.9%). A large majority underwent emergency surgery (61, 87.1%) (Table 3).

**Table 3:** Surgical characteristics This table outlines the surgical profile of patients, including the type of surgery based on anatomical site, procedures performed, and nature of surgery (emergency/elective). Data presentation: Categorical variables are expressed as frequency (n) and percentage (%). Statistical test used: Descriptive statistics only. n – Number of patients, % – Percentage.

Variable	Category	N (%)
Type of Surgery (Anatomical)	Small bowel	29 (41.4%)
	Gastroduodenal	25 (35.7%)
	Large bowel	13 (18.6%)
	Biliary	1 (1.4%)
	Gall bladder	1 (1.4%)
	Others	1 (1.4%)
Procedure Performed	Peptic ulcer repair	14 (20.0%)
	Resection & anastomosis	11 (15.7%)
	Adhesiolysis	9 (12.9%)
	Primary repair	8 (11.4%)
	Trauma (solid organ)	5 (7.1%)
	Pancreatic/cyst procedure	5 (7.1%)
	Sigmoid volvulus surgery	3 (4.3%)
	Hernia repair	2 (2.9%)
	Oncological resection	2 (2.9%)
	Miscellaneous	11 (15.7%)
Nature of Surgery	Emergency	61 (87.1%)
	Elective	9 (12.9%)

Wound dehiscence occurred in 8 (11.4%) patients, whereas wound infection was observed in 29 (41.4%), indicating a higher burden of infectious complications compared to dehiscence (Table 4).

**Table 4:** Postoperative outcomes This table depicts the incidence of postoperative complications, specifically wound dehiscence and wound infection. Data presentation: Data are presented as frequency (n) and percentage (%). Statistical test used: Descriptive statistics only. n – Number of patients, % – Percentage.

Outcome	Category	N (%)
Wound Dehiscence	Present	8 (11.4%)
	Absent	62 (88.6%)
Wound Infection	Present	29 (41.4%)
	Absent	41 (58.6%)

The mean Rotterdam risk score in the study population was 4.40 ± 1.61. Patients with wound dehiscence had significantly higher Rotterdam scores (6.58 ± 0.84) compared to those without (3.95 ± 1.36) (p < 0.001). Similarly, patients with wound infection had higher scores (5.62 ± 1.03) than those without infection (3.27 ± 1.05) (p < 0.001), indicating a strong association of the score for postoperative complications (Table 5).

**Table 5:** Rotterdam score and outcomes This table compares the mean Rotterdam risk scores between patients with and without postoperative complications (wound dehiscence and wound infection). Data presentation: Continuous variables are expressed as mean ± standard deviation (SD). Confidence intervals (95% CI) are also reported. Statistical test used: Independent samples t-test was applied to compare means between groups. SD – Standard Deviation, CI – Confidence Interval, t – t-statistic, p – p-value.

Outcome	Category	Mean ± SD	Test Statistic	95% CI	p-value
Wound Dehiscence	Present	6.58 ± 0.84	t = 5.883	1.75–3.54	<0.001
	Absent	3.95 ± 1.36			
Wound Infection	Present	5.62 ± 1.03	t = 9.268	1.84–2.85	<0.001
	Absent	3.27 ± 1.05			

The Rotterdam score demonstrated excellent predictive accuracy for wound dehiscence with an AUC of 0.956 (p < 0.001), confirming high diagnostic performance. The bootstrap-corrected AUC was 0.89, indicating slight optimism in the apparent model performance (Table 6).

**Table 6:** ROC curve analysis of Rotterdam score This table presents the diagnostic performance of the Rotterdam score in predicting wound dehiscence using receiver operating characteristic (ROC) curve analysis. Data presentation: Results are expressed as area under the curve (AUC), standard error, p-value, and 95% confidence interval (CI). Statistical test used: ROC curve analysis. ROC – Receiver Operating Characteristic, AUC – Area Under the Curve, CI – Confidence Interval, p – p-value.

Parameter	Value
AUC	0.956
Standard Error	0.027
p-value	<0.001
95% CI	0.904–1.000

An optimal cut-off value of ≥5.8 provided the best balance with sensitivity of 90% and specificity of 92%, outperforming lower thresholds. The positive predictive value (PPV) and negative predictive value (NPV) at this cut-off were 59% and 98%, respectively (Table 7).

**Table 7:** Optimal cut-off for Rotterdam score This table shows different cut-off values of the Rotterdam score along with corresponding sensitivity, specificity, and Youden Index to determine the optimal threshold for predicting wound dehiscence. Data presentation: Data are presented as proportions/values. Statistical test used: Derived from ROC analysis (Youden Index calculation). % – Percentage, ROC – Receiver Operating Characteristic.

Cut-off (≥)	Sensitivity	Specificity	Youden Index
4.5	1.00	0.60	0.60
5.4	0.90	0.82	0.72
5.8	0.90	0.92	0.82

Wound dehiscence showed significant associations with COPD (4, 33.3% vs 3, 5.2%, p = 0.003), postoperative cough (2, 100.0% vs 5, 7.4%, p < 0.001), type of surgery (p = 0.049), and wound infection (6, 20.7% vs 1, 2.4%, p = 0.012). Age group, sex, ascites, and jaundice were not significantly associated (p > 0.05) (Table 8).

**Table 8:** Factors associated with wound dehiscence This table evaluates the association between various demographic, clinical, and surgical factors and the occurrence of wound dehiscence. Data presentation: Categorical variables are expressed as frequency (n) and percentage (%). Statistical test used: Fisher's exact test was used to assess associations between variables. n – Number of patients, % – Percentage, χ² – Chi-square test, p – p-value, COPD – Chronic Obstructive Pulmonary Disease.

Variable	Category	Dehiscence Present N (%)	Dehiscence Absent N (%)	p-value
Age Group	18–19	0 (0.0%)	5 (100.0%)	0.116
	20–29	0 (0.0%)	10 (100.0%)	
	30–39	3 (30.0%)	7 (70.0%)	
	40–49	1 (7.1%)	13 (92.9%)	
	50–59	1 (9.1%)	10 (90.9%)	
	60–69	1 (10.0%)	9 (90.0%)	
	70–85	1 (10.0%)	9 (90.0%)	
Sex	Male	3 (7.0%)	40 (93.0%)	0.280
	Female	4 (14.8%)	23 (85.2%)	
COPD	Yes	4 (33.3%)	8 (66.7%)	0.003
Ascites	Yes	1 (20.0%)	4 (80.0%)	0.739
Jaundice	Yes	1 (12.5%)	7 (87.5%)	0.802
Cough	Yes	2 (100.0%)	0 (0.0%)	<0.001
Type of Surgery	Biliary	0 (0.0%)	1 (100.0%)	0.049
	Gall Bladder	1 (100.0%)	0 (0.0%)	
	Gastroduodenal	3 (12.0%)	22 (88.0%)	
	Large Bowel	2 (15.4%)	11 (84.6%)	
	Small Bowel	1 (3.4%)	28 (96.6%)	
	Others	0 (0.0%)	1 (100.0%)	
Wound Infection	Yes	6 (20.7%)	23 (79.3%)	0.012

## Discussion

Abdominal wound dehiscence remains a multifactorial postoperative complication influenced by patient-related, surgical, and postoperative factors. In the present study, age and sex were not significantly associated with wound dehiscence, although the highest proportion was observed in the 30-39 years group (3, 30.0%]) and among females (4, 14.8%]). These findings are consistent with studies by Sadh et al. [13] and Kenig et al. [14], who also reported no significant demographic association. However, other studies have demonstrated contrasting results, with Verma et al. [15] and Kapoor et al. [5] reporting male predominance, and van Ramshorst et al. [9] identifying advanced age and male sex as independent predictors. This variability suggests that demographic factors alone may not reliably predict dehiscence and are likely influenced by population-specific characteristics.

Among systemic factors, anemia was not retained as a significant factor after statistical correction, and therefore, its role should be interpreted with caution. Similarly, pulmonary factors demonstrated a strong influence, with COPD significantly associated with dehiscence (4, 33.3%, p = 0.003), and notably, all patients with postoperative cough developed dehiscence (2, 100.0%, p < 0.001). These findings are consistent with van Ramshorst et al. [9] and Kenig et al. [14], who identified cough and pulmonary disease as major contributors to increased intra-abdominal pressure. Other comorbidities, such as ascites and jaundice, did not show a significant association in the present study, aligning with some reports but differing from studies that identified broader comorbidity burden as a risk factor.

Surgical and postoperative factors played a crucial role in the occurrence of wound dehiscence. A majority of dehiscence cases occurred in emergency surgeries (7 of 8, 87.5%); however, this association was not retained as statistically significant after correction. This is comparable to findings by Sharma et al. [10] and Aregawi et al. [16], where emergency procedures were strongly associated with increased risk. The type of surgery also showed a significant association (p = 0.049), with higher rates observed in gastroduodenal and large bowel procedures, consistent with observations by van Ramshorst et al. [9]. Furthermore, wound infection emerged as a significant postoperative factor, with a higher incidence of dehiscence among infected cases (6, 20.7% vs 1, 2.4%, p = 0.012). However, it is important to note that wound infection is also included as a component of the Rotterdam Risk Index and lies on the causal pathway to dehiscence, introducing potential circularity and overestimation of predictive performance.

The Rotterdam Risk Index demonstrated good discriminative ability in the present study. Patients with wound dehiscence had significantly higher scores compared to those without (6.58 ± 0.84 vs 3.95 ± 1.36, p < 0.001), and ROC analysis revealed an AUC of 0.956, indicating high discriminative ability. However, the bootstrap-corrected AUC was 0.89, suggesting some degree of model optimism. An optimal cut-off value of ≥5.8 yielded 90% sensitivity and 92% specificity. These findings are broadly consistent with those reported by Sharma et al. [10] (AUC 0.986) and Sadh et al. [13], both of which confirmed significantly higher scores among dehiscence cases. The present study differs from Sharma et al. in terms of a higher proportion of emergency surgeries, which may influence both incidence and predictive performance [10]. Kenig et al. also demonstrated the predictive value of the Rotterdam score, although with a relatively lower AUC of 0.76 [14]. The higher AUC observed in the present study may be attributed to the inclusion of postoperative variables in the score, enhancing apparent discrimination. Overall, these findings support the association and discriminative ability of the Rotterdam Risk Index within this cohort rather than its role as a purely predictive tool.

Strengths and limitations

This study has several strengths, including its prospective design, standardized data collection, and comprehensive evaluation of multiple preoperative, intraoperative, and postoperative risk factors in a real-world surgical setting. The use of the Rotterdam Risk Index and its validation via ROC analysis adds significant clinical relevance in terms of assessing discriminative performance.

However, certain limitations must be acknowledged. The relatively small sample size (n = 70) and single-center design may limit the generalizability of the findings. Additionally, the predominance of emergency surgeries may have introduced selection bias and influenced the risk profile. Only bivariate analysis was performed without multivariate adjustment, limiting the identification of independent predictors. Patients undergoing relaparotomy were excluded, which may limit representation of higher-risk populations. The Rotterdam Risk Index includes postoperative variables such as wound infection and cough, which lie on the causal pathway of dehiscence, introducing circularity and limiting its use as a purely predictive model. Some potentially important variables, such as nutritional status, BMI, and smoking history, were not assessed, and longer follow-up beyond the early postoperative period was not performed. External validation in larger and diverse populations is required, and the development of a purely preoperative risk model may provide greater clinical utility.

## Conclusions

Abdominal wound dehiscence remains a significant postoperative complication influenced by a combination of patient-related, surgical, and postoperative factors. The present study demonstrates that COPD, postoperative cough, type of surgery, and wound infection are important contributors to its occurrence, while demographic factors showed no significant association. The Rotterdam Risk Index demonstrated good discriminative ability in this cohort. However, as the index incorporates postoperative variables, it reflects association rather than early prediction. Further studies with larger sample sizes, multivariate analysis, and external validation are required before routine clinical application. Development of a purely preoperative risk model may provide greater clinical utility.
